# Dental caries in the peruvian police population

**DOI:** 10.4317/jced.54265

**Published:** 2018-02-01

**Authors:** Luz A. Moreno-Quispe, Luis A. Espinoza-Espinoza, Loel S. Bedon-Pajuelo, Magna Guzmán-Avalos

**Affiliations:** 1 MS, DDS, Researcher, Department of Research, Innovation and Development Peru SAC, Ancash Peru; 2 MS, Researcher, Department of Research, Innovation and Development Peru SAC, Ancash Peru; 3 MS, Ph.D, Professor, Social Policies Program, Graduate School, Santiago Antúnez de Mayolo University, Ancash Peru; 4 MS, Ph.D, Professor, School of Health Sciences, Santiago Antúnez de Mayolo University, Ancash Peru

## Abstract

**Background:**

Dental caries is a multifactorial disease that affects the general population. After reviewing the scientific literature, no studies were found on the index of decayed, missing and filled teeth (DMFT) in the Peruvian police population. The objective was to evaluate the DMFT index and severity level of the disease in police personnel of the Ancash region, Peru.

**Material and Methods:**

Cross-sectional prevalence study. The medical records of the police personnel in activity were reviewed and each subject was examined from May 2012 to May 2013. The study was authorized by the Director of the PNP-Huaraz Ancash Polyclinic as part of the activities of the civil SERUMS personnel in the area of odontology. The sample was census with 925 subjects. The data was systematized following the methodology recommended by the World Health Organization (WHO). The statistics were analyzed by Chi square test with significance *p*<0.05, Pearson test and ANOVA.

**Results:**

The prevalence of caries in the police population was 73.4%. The DMFT index was 10.63 ± 4.96 (*p*<0.01). The severity of the disease in relation to age was 0.77 ± 0.41 with a high risk in this population. The DMFT index in females 128/925 and males 797/925 was 10.43 and 10.67 respectively. There is an inversely proportional relationship in the number of teeth filled with dental amalgam in policemen older than 35 years versus the number of teeth sealed with material other than dental amalgam in policemen under 35 years. Only 0.8% 7/925 had dental prostheses and 58.6% (542/925) of the subjects needed oral rehabilitation.

**Conclusions:**

The severity of dental caries is high, strategies are required to improve intervention in this sector, developing effective programs in oral health in the short, medium and long term.

** Key words:**Dental caries severity, oral health, dental caries prevalence, peruvian police.

## Introduction

Dental caries is a chronic medical pathology of multifactorial origin that demineralizes the tissue of the tooth until the formation of a cavity if it is not treated in a timely manner (1-3).

The disease affects the general health and quality of life of people of all ages (4-6). In recent years little attention has been paid to the prevalence of dental caries in the Peruvian population, especially the police, despite the emergence of health management instruments in the country, whose national health system is made up of the Ministry of Health, Social Security Essalud, Health of the armed forces and police (PNP) and the private health service.

The health of the police and the armed forces is in charge of the budget and policies of the Ministry of Interior and Defense, due to this there is an oral health program led by the PNP National Health Directorate and each year they call for civilian dentists to perform the marginal urban service (SERUMS) in PNP Health Policlinics located in geographical areas of the interior of the country.

In Peru, health professionals can only work in the public health system at the end of SERUMS. The budget for SERUMS dentists is limited, the salary assigned to this group of professionals is the minimum compared to workers in other sectors of the Peruvian State; even when it develops multiple activities: in the clinical-assistance, administrative, preventive-promotional, teaching and research field. Nevertheless, the experience and the good practices that the odontologists acquire in this professional stage is undeniable. For this reason, the present study had the objective of evaluating the DMFT index and the level of severity of the disease in the police personnel of the Ancash region, Peru, in order to have scientific evidence to improve the oral health program of the Polyclinic.

## Material and Methods

The city of Huaraz is home to police sanitation in the Ancash region whose PNP Policlinic Huaraz has the Department of Dentistry, where the study was conducted from May 2012 to May 2013. The inclusion criteria of the study were; a) policemen in activity, b) policemen who complete the annual medical examination and c) over 18 years of age. The exclusion criteria were; a) relatives of police personnel receiving dental service, b) police in retirement and c) police personnel in training receiving dental treatment.

A population of 925 active policemen were registered and examined, who were included in the research. Therefore, the census sample was of 925 subjects. Authorization was requested to start the study as part of the activities of the dental staff SERUMS and was approved by the Director of the Policlynic PNP Huaraz. The SERUMS civilian personnel in charge of the study was authorized to work through Directorial Resolution N° 588-2012-DIRGEN/DIRREHUM.

The patients signed an informed consent. Patient data and informed consent were treated in compliance with the Helsinki declaration and subsequent revisions, as well as the legal regulations applicable in Peru based on the characteristics of the present study.

**Figure 1:**
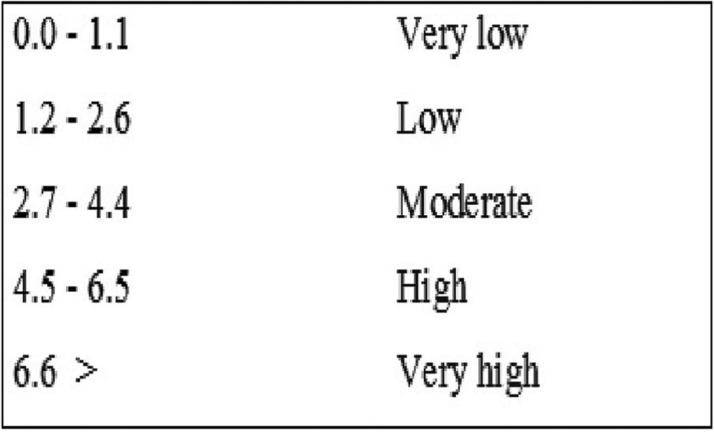
WHO quantification for the DMFT index.

This is a cross-sectional prevalence study. The examiners were trained and calibrated. The data was collected and systematized following the methods and criteria of the WHO, 1997(7). Patients were distributed by age groups of 18-24 years, 25-34, 35-44 and 45-64 respectively (8). The quantification of the severity of dental caries was defined according to WHO parameters, 1986 (Fig. 1).

The statistic was analyzed using the SPSS version 20.0 for Windows using the Chi square test, binomial test and Kruskal-Wallis for independent samples with significance level of *p* <0.05. To relate the variables, the unilateral Pearson correlation was used and in the comparison of means, ANOVA test was used.

## Results

-Dental caries in relation to the age of the study population

Of all subjects, 19% (174/925) were between 18-24 years of age, 40% (369/925) between 25-34 years, 20% (185/925) between 35-44 years and 21% (197/925) aged 45-64 years. The mean and standard deviation of the DMFT index of the total of subjects was (10.63 ± 4.96). The number of teeth presented with caries was = 1625 and (1.75 ± 1.51), missing n = 1437 and (1.55 ± 2.15), filled up n = 6775 and (7.32 ± 4.51) adding a total of exposed teeth n = 9837 ( Table 1). Subjects with ages ranging from 45-64 years had a higher score of DMFT (11.88 ± 5.22) in relation to other age groups.

**Table 1:**
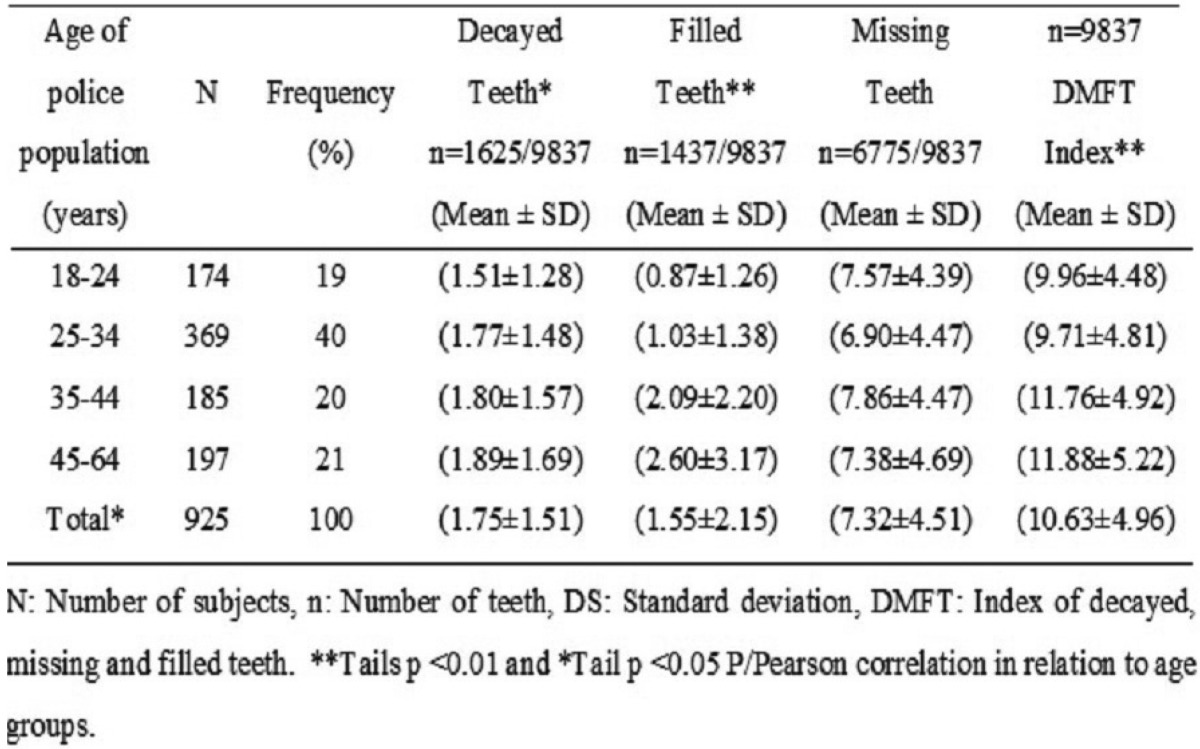
Distribution of the DMFT index according to age groups.

The severity of tooth decay in relation to the age of the population was very low, 1.7% (16/925), 2.1% (20/925), moderate 7% (65/925), high 88% (818/925) and very high 77.5% (717/925). Analyzing the DMFT index variables with the patient’s age, a score above the mean ± SD was observed in the “very high” severity level in all age groups with a significant correlation in ** tails with the test of P / Pearson *p* <0.01 ( Table 2). Among the variables we also observed the “very low” severity level that belonged to the age group of 18-24 years old, unlike other groups with a P * Pearson tail *p* <0.05.

**Table 2:**
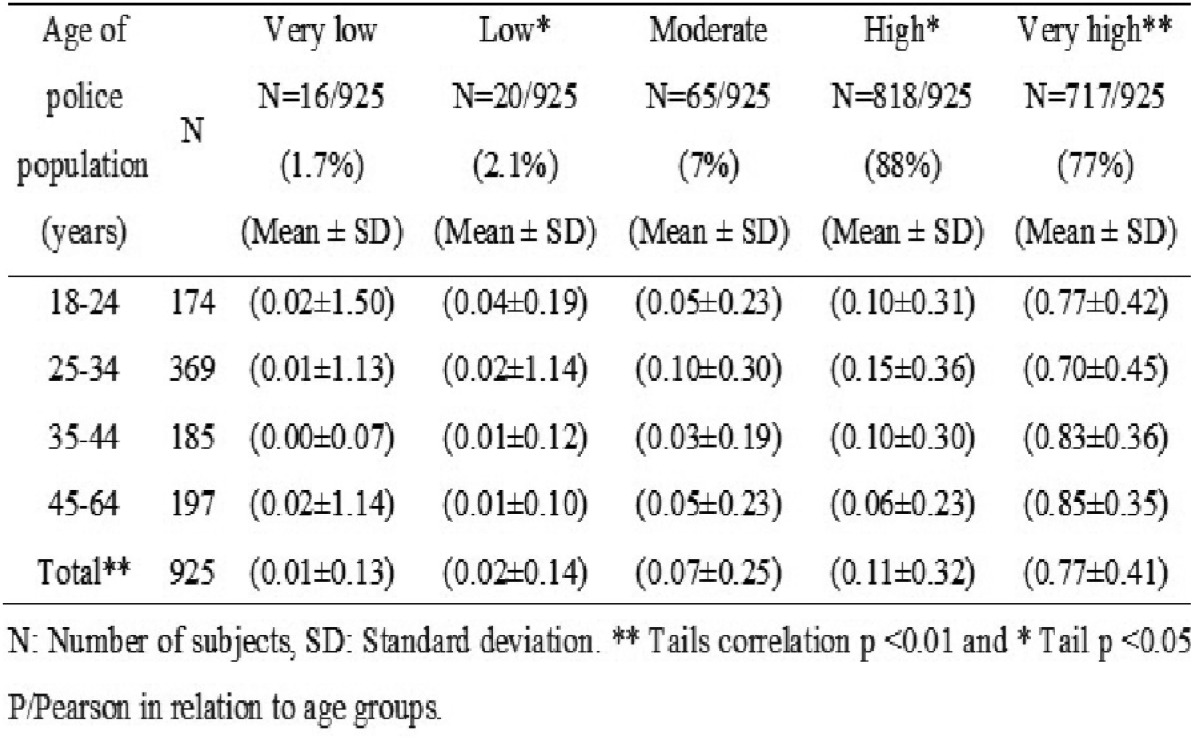
Distribution of the severety level of dental caries according to age groups.

Of the 925 subjects, 86.2% (797/925) of the male gender and 13.8% (128/925) of the female gender were observed ( Table 3). The DMFT index in both groups was 10.67 and 10.43 respectively, although there were less subjects in the female group, the male group had a very high index of dental caries.

**Table 3:**
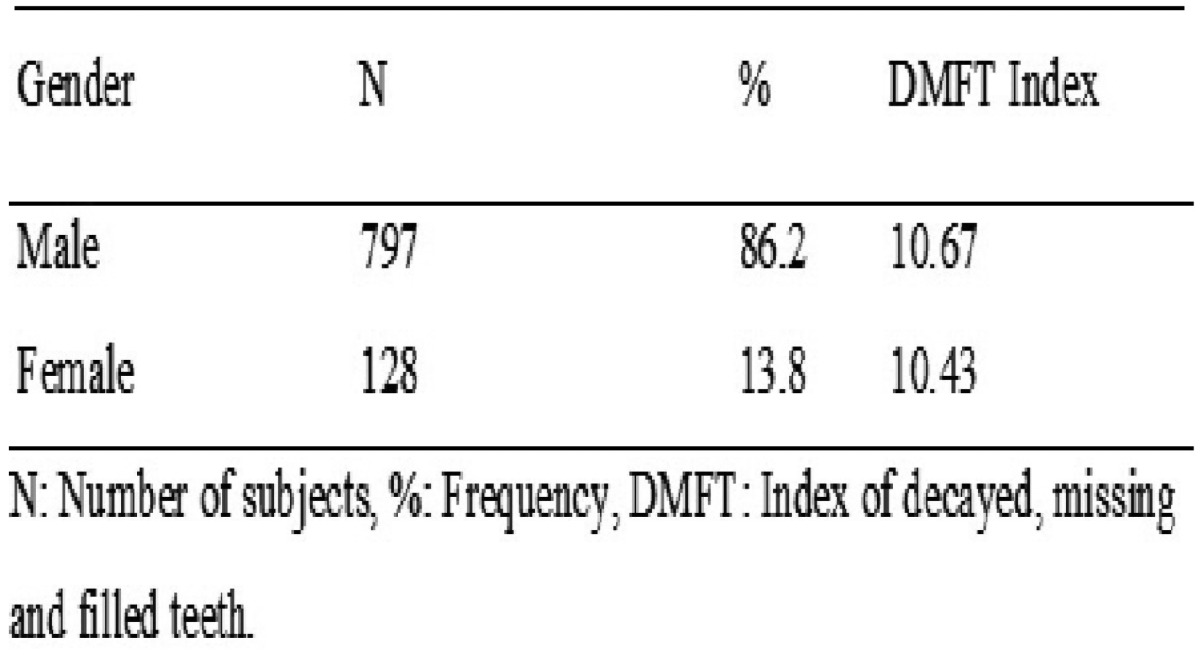
DMFT index according to gender distribution.

Dental fillings according to type of restorative material

The 925 subjects had a total of 6775 obturated teeth, of which 53% (3599/6775) were teeth filled with dental amalgam and 47% (3176/6775) with a material other than dental amalgam ( Table 4). It was observed that the age group 35-44 years had a mean ± SD (4.87 ± 4.34) of teeth with dental amalgam that increased as the age advanced, but when compared with the mean ± SD of teeth with non-amalgam restorations of the same age group, there were fewer (2.98 ± 3.63) amalgam restorations, even when the population was younger. This data suggests that the young population of less 35 years preferred to have a different restoration to dental amalgam, although the health dentistry service PNP Huaraz in these years has had a continuous supply of dental amalgam, although from 2013 there were modifications in the order of dental restorative material, opting for restorative materials other than dental amalgam.

**Table 4:**
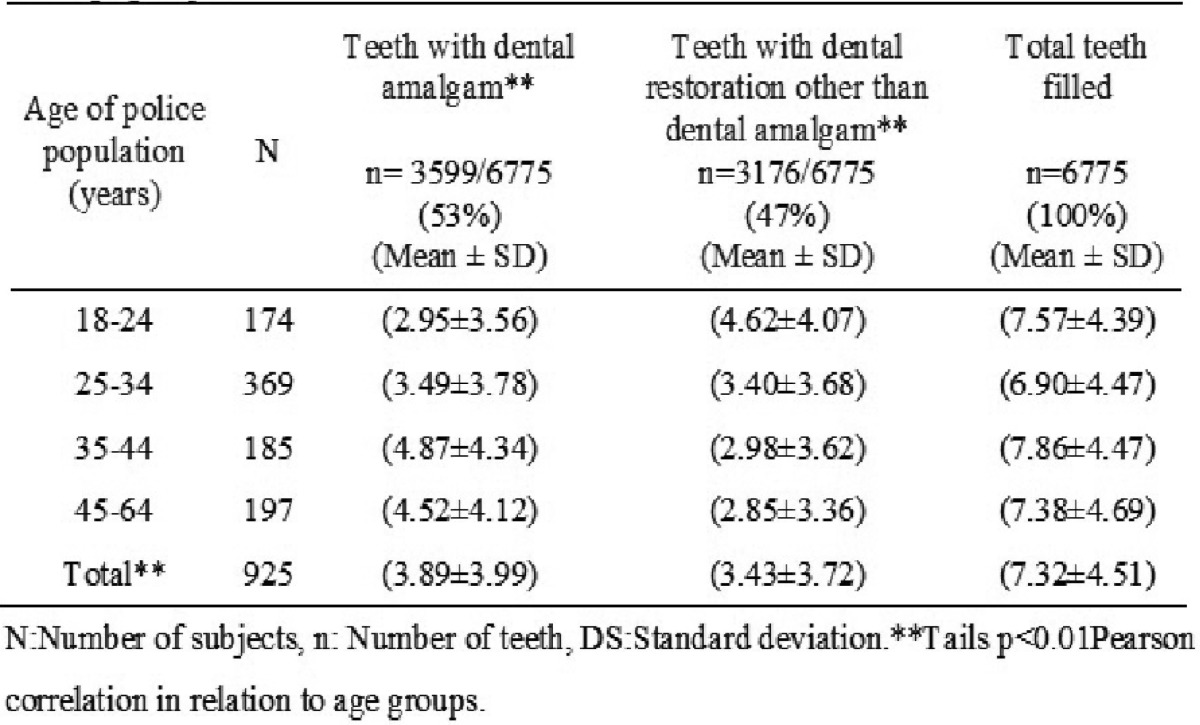
Distribution of the number of teeth according to dental restorative material type and age groups.

-Dental prosthesis in the study population

Of the total of subjects only 0.8% (7/925) used removable prosthesis and these were in poor condition. The 58.6% (542/925) presents the need for oral rehabilitation due to tooth loss. The age groups 35-44 years and 45-64 years had a mean ± SD of upper teeth lost in relation to other age groups (0.73 ± 0.44), who required oral rehabilitation.

## Discussion

The purpose of the study was to provide scientific evidence to improve the oral health program of the Huaraz PNP Polyclinic; therefore, the study was started by evaluating the DMFT index only of the police personnel who met the inclusion criteria. Police officers in retirement, police in formation or relatives of police did not participate, who also receive services of dentistry of the PNP Huaraz Polyclinic.

The research has been limited to know the DMFT index and its relation with the population of study by age groups. No data has been collected on their socioeconomic level, quality of life, oral hygiene habits, fluoridation treatment, type of diet, type of sugar intake and frequency of consumption per day. Therefore, we cannot value higher contributions offered by PNP Huaraz Polyclinic dentistry service. However, the results of this report serve the competent authorities as a background to the search for effective strategies to reduce the rate of caries in the police population.

The dental caries index collected in the study was “very high” for all age groups and these data coincide with other investigations conducted in the police or military population (8-10).

In the study, the number of women was lower than that of men, a similar finding was reported by Farago *et al.*, Abhishek *et al.* and Hopcraft *et al.* (7,8,10). However, in our study, both groups show similar values for the caries index 10.43 vs 10.67, coincident with studies by Faragó et al. and Hopcraft *et al.* (7,11). This data suggests a review of the oral hygiene habits, type and frequency of diet consumed by the subjects and the number of dental visits that would be necessary to program the reduce the high risk of dental caries. In addition, especially women during their maternal stage in relation to their children generate a “window of infectivity”, that is to say they produce greater cariogenic susceptibility in the process of ontogeny of their son transmitting Streptococcus Mutans by means of the saliva and crevicular fluid p.e. through the blowing on of the meals and the use of the same spoon to feed the child in the early stages of life (6,12).

Our results show that 53% (3599/6775) of the total teeth presented restorations with dental amalgam and 47% (3176/6775) with dental material other than amalgam. These data coincide with studies conducted by Kingman *et al.* on high numbers of dental surfaces exposed to dental amalgam in the military population (13). However, there are several studies that explain the release of mercury vapor that comes from restorations with dental amalgam detected in the placement process and during its removal (13-16). Mercury vapors have also been identified during polishing, chewing and corrosion of dental amalgam restorations in patients (17,18).

However, the data found in our study may vary over time, because on October 10, 2013, Peru signed the Minamata Convention whose objective is to protect human health and the environment from anthropogenic releases and releases of Mercury and its compounds, approved and ratified by Legislative Resolution No. 30352 and Supreme Decree No. 061- 2015-RE. This legal regulation allows an improvement in the national management of mercury and all use of this chemical element and its derivatives in the Country.

The data collected on the number of subjects using dental prosthesis coincide with the results of the study by Ahuja *et al.* (19), although we did not collect more information about the type of prosthesis in the maxilla or jaw as reported by Lo EC *et al.* (20). Our research reports the number of missing teeth that are needed to rehabilitate them to improve the masticatory capacity of the police.

In conclusion, the present study demonstrated that there is a high DMFT index in the police population, and it is recommended to propose more effective strategies in the oral health program to reduce the indicators of dental caries in these subjects.
